# High level of aneuploidy and recurrent loss of chromosome 11 as relevant features of somatotroph pituitary tumors

**DOI:** 10.1186/s12967-024-05736-0

**Published:** 2024-11-04

**Authors:** Julia Rymuza, Paulina Kober, Maria Maksymowicz, Aleksandra Nyc, Beata J. Mossakowska, Renata Woroniecka, Natalia Maławska, Beata Grygalewicz, Szymon Baluszek, Grzegorz Zieliński, Jacek Kunicki, Mateusz Bujko

**Affiliations:** 1 Department of Molecular and Translational Oncology, Maria Sklodowska-Curie National Research Institute of Oncology, Warsaw, Poland; 2 Department of Cancer Pathomorphology, Maria Sklodowska-Curie National Research Institute of Oncology, Warsaw, Poland; 3 Cytogenetic Laboratory, Maria Sklodowska-Curie National Research Institute of Oncology, Warsaw, Poland; 4 Department of Neurosurgery, Military Institute of Medicine, National Institute of Medicine, Warsaw, Poland; 5 Department of Neurosurgery, Maria Sklodowska-Curie National Research Institute of Oncology, Warsaw, Poland

**Keywords:** Acromegaly, Pituitary tumors, Growth hormone-secreting pituitary adenoma, DNA copy number variations, Cytogenetic abnormalities, Gene expression regulation, neoplastic

## Abstract

**Background:**

Somatotroph neuroendocrine pituitary tumors (sPitNET) are a subtype of pituitary tumors that commonly cause acromegaly. Our study aimed to determine the spectrum of DNA copy number abnormalities (CNAs) in sPitNETs and their relevance.

**Methods:**

A landscape of CNAs in sPitNETs was determined using combined whole-genome approaches involving low-pass whole genome sequencing and SNP microarrays. Fluorescent in situ hybridization (FISH) was used for microscopic validation of CNAs. The tumors were also subjected to transcriptome and DNA methylation analyses with RNAseq and microarrays, respectively.

**Results:**

We observed a wide spectrum of cytogenetic changes ranging from multiple deletions, recurrent chromosome 11 loss, stable genomes, to duplication of the majority of the chromosomes. The identified CNAs were confirmed with FISH. sPitNETs with multiple duplications were characterized by intratumoral heterogeneity in chromosome number variation in individual tumor cells, as determined with FISH. These tumors were separate CNA-related sPitNET subtype in clustering analyses with CNA signature specific for whole genome doubling-related etiology. This subtype encompassed *GNAS*-wild type, mostly densely granulated tumors with favorable expression level of known prognosis-related genes, notably enriched with *POUF1*/*NR5A1*-double positive PitNETs. Chromosomal deletions in sPitNETs are functionally relevant. They occurred in gene-dense DNA regions and were related to genes downregulation and increased DNA methylation in the CpG island and promoter regions in the affected regions. Recurrent loss of chromosome 11 was reflected by lowered *MEN1* and *AIP.* No such unequivocal relevance was found for chromosomal gains. Comparisons of transcriptomes of selected most cytogenetically stable sPitNETs with tumors with recurrent loss of chromosome 11 showed upregulation of processes related to gene dosage compensation mechanism in tumors with deletion. Comparison of stable tumors with those with multiple duplications showed upregulation of processes related to mitotic spindle, DNA repair, and chromatin organization. Both comparisons showed upregulation of the processes related to immune infiltration in cytogenetically stable tumors and deconvolution of DNA methylation data indicated a higher content of specified immune cells and lower tumor purity in these tumors.

**Conclusions:**

sPitNETs fall into three relevant cytogenetic groups: highly aneuploid tumors characterized by known prognostically favorable features and low aneuploidy tumors including specific subtype with chromosome 11 loss.

**Supplementary Information:**

The online version contains supplementary material available at 10.1186/s12967-024-05736-0.

## Background

Somatotroph neuroendocrine pituitary tumors (sPitNETs) are a subtype of the neoplasms of the anterior pituitary lobe. These tumors are commonly clinically hormonally functioning since they secrete high levels of growth hormone (GH) that result in increased level of circulating insulin-like growth factor-1 (IGF-1). Somatotroph PitNETs are a major cause of acromegaly, a disease manifested by the overgrowth of certain parts of the body: hands and feet, changes in facial features, hypertrichosis, macroglossia, and complications such as diabetes, hypertension, cardiomyopathy, osteoarthritis, hypogonadism, colon polyps, and nerve entrapment syndromes [1].

Pituitary tumors that cause acromegaly are relatively heterogeneous. They vary in radiological image, pathomorphological characteristic, and molecular features [2, 3]. The somatotroph origin of these PitNETs is determined by histological examination that includes immunohistochemical staining for GH and PIT-1 transcription factor. Histologically, they can be divided into sparsely and densely granulated (SG and DG, respectively) with electron microscopy-based evaluation or anti-cytokeratin staining [4]. The spectrum of PitNETs that cause acromegaly includes basic tumors that produce GH but also tumors that express additional pituitary hormones such as mammosomatotroph or mature plurihormonal tumors, and other rare subtypes distinguished according to the WHO classification of PitNETs [5].

The best recognized tumor-driving molecular changes in somatotroph PitNETs are activating mutations in the *GNAS* gene. This gene encodes a heterotrimeric G protein stimulatory subunit that is crucial for activation of the cAMP pathway following stimulation of the GHRH receptor, which is the main stimulatory element in the hypothalamus-pituitary axis. Somatic activating *GNAS* mutations are observed in 40% of somatotroph tumors and, in general, they are the only highly recurrent point mutation in tumors causing acromegaly [6]. In contrast to low level of point mutations, there is a much higher load of structural cytogenetic changes in somatotroph PitNETs. Throughout the years, DNA copy number abnormalities (CNAs) have been identified in this type of tumors with various laboratory methods that use microsatellite markers, comparative genomic hybridization, microarrays based on single nucleotide polymorphism (SNP) and DNA methylation, and sequencing based methods [7–18]. These studies did not provide a complete consensus overview of the spectrum of cytogenetic changes in somatotroph PitNETs as an inconsistency can be clearly noted between the results. In our study, we made an attempt to describe the landscape of structural changes in these tumors with two different complement genome-wide methods in sPitNETs and to determine the role of these abnormalities in tumorigenesis.

## Methods

### Patients and tissue samples

Forty tumor tissue samples were included from patients with biochemically confirmed acromegaly. The patients were treated with transsphenoidal surgery in the Department of Neurosurgery, Military Institute of Medicine, Warsaw and the Department of Neurosurgery, Maria Sklodowska-Curie National Research Institute of Oncology, Warsaw, between 2013 and 2020. Diagnostic criteria for acromegaly were based on clinical and biochemical characteristics, including increased serum IGF-1 levels and nonsuppressible GH after Oral Glucose Tolerance Test (OGTT), in patients without contraindications to OGTT. All patients were treated with somatostatin receptor ligands (octreotide or lanreotide) before surgery, following the recommendations of the Polish Society of Endocrinology [21].

Histopathological evaluation included both immunohistochemical staining and ultrastructural analysis with electron microscopy. Evaluation of the immunoexpression of pituitary hormones (GH, PRL, ACTH, TSH, FSH, LH, α-subunit) and Ki-67 was performed for diagnosis. The immunohistochemical expression of the PIT-1 transcription factor was retrospectively confirmed in all tumors, as a large proportion of tumors was originally diagnosed according to WHO 2014 criteria. All included tumors were ‘pure’ somatotroph PitNETs that express GH and PIT-1, without the expression of other pituitary hormones. The ultrastructural status (sparsely vs. densely granulated tumors) was determined by electron microscopy. The general characteristics are presented in Table 1.

**Table 1:** Summary of demographic and clinical features of patients with acromegaly

Clinical Feature	
**Number of patients**	*n* = 40
**Sex**	
Females	25/40 (62.5%)
Males	15/40 (37.5%)
**Age at surgery** (years; median (range))	40 (22–74)
**GH** (µg/dL; median (range))	10.1 (0.89–171)
**IGF-1** (µg/dL; median (range))	704.5 (166–1600)
**Tumor size -** max. diameter (mm; median (range))	18 (5.1–77)
**Invasive tumor growth**	
Invasive tumors (Knosp grade III, IV)	15/40 (37.5%)
Noninvasive tumors (Knosp grade 0, I, II)	25/40 (62.5%)
**Granulation pattern**	
DG somatotroph tumors	23/40 (57.5%)
SG somatotroph tumors	17/40 (42.5%)
***GNAS*** **mutation**	8/40 (20%)

Invasive growth was determined on preoperative magnetic resonance imaging using Knosp classification. Tumors scored with Knosp grades 0–2 were considered non-invasive, while those with Knosp grades 3–4 were considered invasive [19].

The study protocol was approved by the local Ethics Committee of the Maria Sklodowska-Curie National Institute of Oncology in Warsaw, Poland. Each patient provided their informed consent for the use of tissue samples for scientific purposes.

DNA and total RNA from tumor samples were isolated with the AllPrep DNA/RNA/miRNA Universal Kit (QIAGEN) and stored at -70 °C. All tumors were tested for *GNAS* mutations (exons 7 and 8) as previously reported [20]. The study included 32 *GNAS* wild-type and eight *GNAS*-mutated somatotroph PitNETs.

### Whole genome copy number analysis

#### Low-pass whole genome sequencing

The results of whole genome sequencing (WGS) with at least x5 coverage were used to analyze the cytogenetic status in 40 tumor samples. The TruSeq DNA Nano kit (Illumina) was used for library preparation and pair-end (2 × 150 bp) sequencing was performed using the NovaSeq 6000 instrument (Illumina). Sequencing was provided by the CeGaT GmbH (Tübingen, Germany) service. Raw reads from WGS were subjected to quality control using FastQC [http://www.bioinformatics.babraham.ac.uk/projects/fastqc] and mapped to genome assembly hg19 using SpeedSeq [21]. Copy number alterations were called using CNVpytor [22] with bin size 10,000. Additional manual adjustment for samples with ploidy greater than two was done. The analysis was limited to autosomes. CNAs from problematic regions were excluded based on the ENCODE blacklist and the UCSC Unusual Regions tracks from the UCSC Genome Browser [23].

#### Copy number analysis with SNP genotyping oligonucleotide microarrays

Illumina’s beadChip technology CytoSNP-850 K genotyping oligonucleotide microarrays (Illumina) were used for DNA copy number profiling. This array covers ~ 850,000 empirically selected single nucleotide polymorphisms (SNPs) spanning the entire genome with enriched coverage for 3,262 genes of known cytogenetic relevance in both constitutional and cancer applications (selected based on data from the Cancer Genomics Consortium). DNA samples were hybridized to CytoSNP-850 K arrays (Illumina) following the suppliers’ recommendations and scanned using the iScan System (Illumina). The original iScan files were analyzed with Illumina GenomeStudio software. The DNA hybridization and scanning of the arrays were performed by the Eurofins Genomics service.

CNAs were called from GenomeStudio (Illumina) results using both Genome Alteration Print (GAP) [24] and genoCN [25]. The results of the tools were integrated using bedtools intersection operation [26] to get high quality CNA. To integrate the results of both the SNP array and the WGS, they were merged using the bedtools merger operation [26]. All acquired alterations were visually inspected by plotting the read depth of the WGS, and the B allele frequency (BAF) and the signal intensity logR ratio (LRR) from the SNP array (Supplementary Fig. 1). For further analysis, the resulting regions were mapped to chromosome bands if they overlapped 60% of a band. The quality of using chromosome bands as the representation of CNA was assessed using F_10_ score and Region Boundary Score (RBD) from the geniml package [27]. These transformed data were used for sample visualization; unsupervised hierarchical clustering using the binary distance and Ward agglomeration method; and detection of recurrent variants. The statistical analysis to detect recurrent CNA for all samples as well as detected clusters was performed using the Genomic Identification of Significant Targets in Cancer (GISTIC) method [28] implemented in CNVRanger R package [29]. CNA signatures for each cluster were calculated and the most similar COSMIC signature (https://cancer.sanger.ac.uk/signatures/cn/) [30] was chosen based on Euclidiean similarity of signatures vector representations. Previously calculated copy number patterns of 10,674 TCGA samples were used to compare the CNAs pattern between sPitNEt and others cancer types [30]. For each sample, aneuploidy score (AS) was calculated. AS was defined as a number of chromosome arms covered in 80% by CNA.

As an external validation of our results, we analyzed the CNA from previous study [8]. We mapped CNA to the chromosome bands and performed hierarchical clustering of the samples with the cosine distance and Ward agglomeration method. Additionally, we used provided transcripts counts (E-MTAB-7768, Array Express) to assess expression level of *NR5A1* in the samples using counts per million (CMP).

### Fluorescence hybridization in situ

Fluorescence hybridization in situ (FISH) analysis was performed on formalin-fixed paraffin-embedded (FFPE) tumors. FFPE specimens were prepared with a Pretreatment Reagent Kit (Wuhan Healthcare Biotechnology, Wuhan, Hubei, China) according to the manufacturer’s protocol. For the confirmation of gains of chromosomes 5, 8, 14, 20, the following probes were used: EGR1 /D5S721/D5S23, MYC breakapart (BAP), IGH BAP, D20S108 (Vysis Abbott Molecular, Downers Grove, IL, USA). For the verification of the losses of chromosomes 1p, 11, 15q, the following probes were applied: 1p36/1q25 (Zytovision GmbH Bremerhaven, Germany), CCND1 BAP, PML/RARA Dual Fusion (Vysis Abbott Molecular). The FISH results were analyzed using a fluorescence microscope, Axioskop2 (Carl Zeiss, Jena, Germany), documented by the BioView system (Abbott Molecular, Abbott Park, IL, USA). Depending on the probe, a cut-off point was established as 15–20% for deletions and 5–10% for gains.

#### Whole transcriptome sequencing

RNA sequencing with Illumina technology was applied for whole transcriptome analysis as previously described [20]. Briefly, 1 µg RNA from each sample was used for library preparation using the NEBNext Ultra II Directional RNA Library Prep Kit and the NEBNext rRNA Depletion Kit. Subsequently, 150-bp pair-end reads were generated with the Illumina NovaSeq 6000 platform. A minimum of 30 M read pairs per sample were generated. RNA-Seq data are available at ArrayExpress: E-MTAB-11889. The data analysis was done using a previously established pipeline [20]. The quality control of the raw reads was performed using FastQC [31] followed by mapping to the human reference genome hg19 with HISAT2 [16]. The transcript counts were calculated with featureCounts [32]. Differential gene expression analysis was performed with DESeq2 [33] and gene set enrichment analysis (GSEA) was conducted with fgsea [34]. The expression level of the cytogenetic bands was calculated using the R library GSEAlm [10.18129/B9.bioc.GSEAlm]. The copy number of a given gene was estimated as an average of the copy number of the genome location detected by GAP, genoCN, and CNVpytor.

#### DNA methylation and copy number profiling with EPIC microarrays

Infinium MethylationEPIC BeadChip Array (Illumina) was used to profile whole-genome DNA methylation in all samples, as described previously [35]. EPIC array data is available at Gene Expression Omnibus: GSE226764. The Minfi R library was used for data analysis [36].

## Results

### The landscape of cytogenetic changes in somatotroph pituitary tumors

**Fig. 1:**
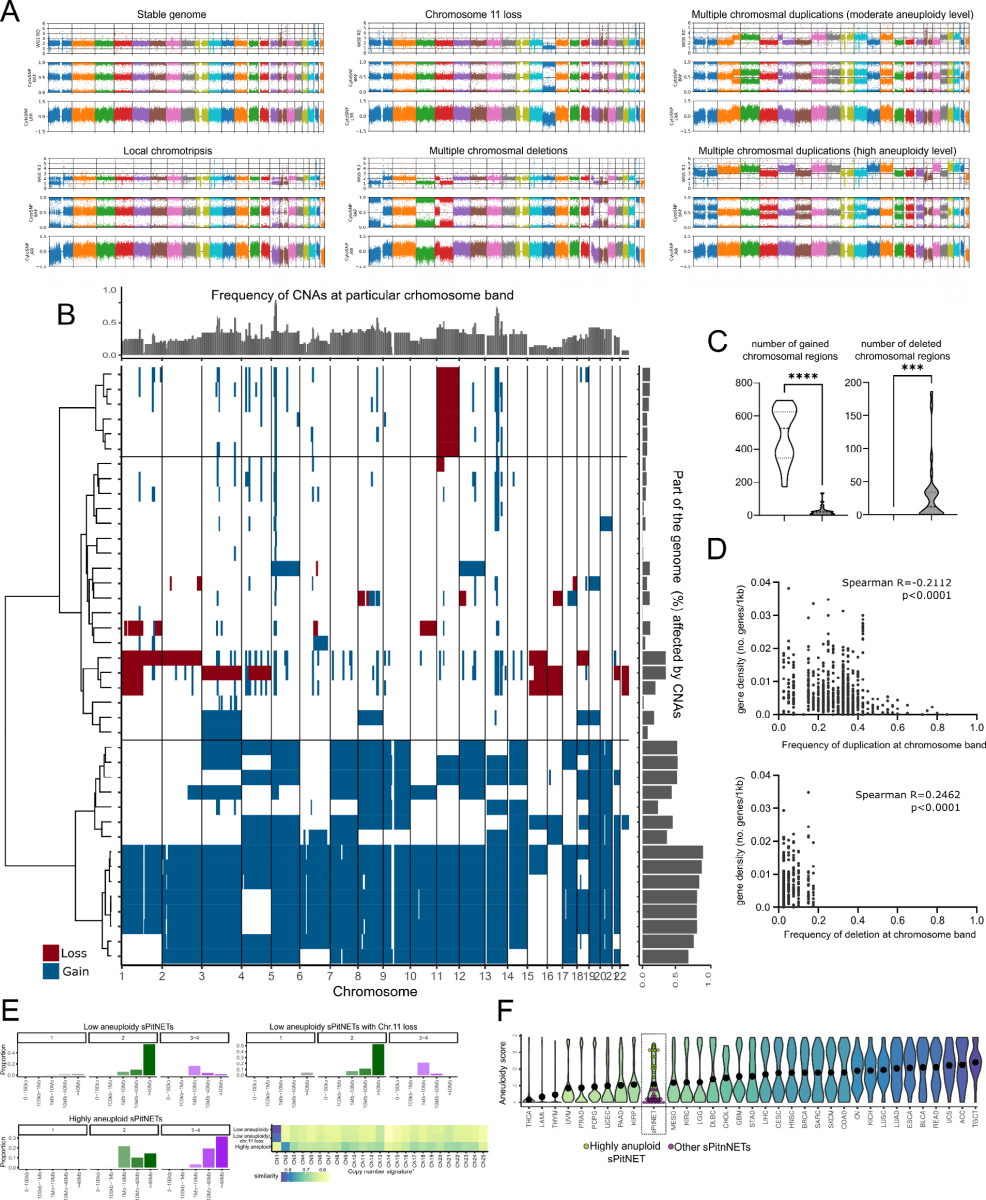
Genome-wide DNA copy number profile in somatotroph pituitary tumors. **A**) Representative examples of virtual karyotypes with copy number patterns observed in the study, based on low pass WGS coverage (WGS RD line), SNP biallelic frequency (CystoSNP BAF line), and microarray probe signal (CystoSNP LRR line); **B**) Landscape of copy number alteration (CNAs) in sPitNETs with a resolution of single chromosomal bands. The dendrograms based on similarity in the CNAs pattern indicate two main cytogenetic clusters of the tumors; **C**) Comparison of the number of chromosomal deletions and duplications in two major cytogenetic clusters of tumors. **D**) Correlation between frequency of incidence of the duplications and deletions at particular chromosomal regions (bands) and gene density; **E**) CNA signatures for each of the three somatotroph tumor clusters and their comparison to known CNA signatures found in human cancers [30] by measuring Euclidiean similarity; **F**) The comparison of CNAs profile in sPitNEts and human cancers included in TCGA project

Both WGS and SNP arrays were used independently to determine the CNAs pattern in each tumor sample. These two approaches showed concordant results and allowed inclusion of three information layers (sequencing reads depth, microarray signal intensity, and B-allele frequency) to conclude a combined cytogenetic landscape in each sample. The analysis of 40 somatotroph PitNETs showed a wide spectrum of chromosomal abnormalities including duplications of multiple chromosomes/chromosomal arms, deletions of multiple chromosomal arms, deletion of single chromosomes, chromothripsis-type chromosome shattering, and relatively stable genomes with only few local DNA CNAs. Virtual karyotype examples for representative samples are shown in Fig. 1A, while all samples are shown in Supplementary Fig. 1. Individual CNA patterns were mapped to chromosomal bands, which represented underlying data well (F_10_ = 0.91, RBS < 0.0001), to generate a comprehensive profile of the copy number changes in the study group (Fig. 1B). The numbers of the identified DNA copy number changes per sample ranged between 1 and 695, the coverage of individual genome by CNAs ranged between 0 and 0.85, while aneuploidy score was between 0 and 35. These 3 measures of genomic instability were highly correlated (Pearson *R* > 0.99, *p* < 0.0001). Chromosomal gains were much more common than deletions. A mean of 199.6 individual duplications per sample was found, compared to 19.05 mean deletions per sample (median 32 duplications vs. 0 losses per sample). The group of patients was significantly diverse in terms of the number of CNAs identified in the tumor sample.

The frequency of CNAs of a particular chromosomal region (chromosomal band) in patients ranged between 0 and 85%. Significantly recurrent CNAs according to GISTIC method were gains of chromosome 5p12, 5p13, 5p14, 5p15, as well as chromosome 13q12, 13q13, 13q14, 13q21, 13q22, 13q31, 13q32, 13q33, 13q34.

We found that the frequency of chromosomal losses was positively correlated with gene density (Spearman *R *= 0.2462; *p *< 0.0001), while the frequency of chromosomal gains was negatively correlated with genes density (Spearman *R*=-0.2112; *p* < 0.0001). Especially, the regions that are duplicated in more than 50% of the samples are generally the regions with no gene or low genes density, as indicated in Fig. 1D. Thus, in sPitNETs chromosomal losses are relatively rare, but they occur in gene-enriched genomic regions, opposite to chromosomal duplications that are much more common but affect regions with lower gene content. This suggests that chromosomal gains, especially those highly recurrent, are rather functionally neutral, while recurrent chromosomal deletions are more relevant.

The unsupervised hierarchical clustering of the samples according to the similarity of the CNAs pattern showed two major clusters of tumors that notably differ in the scale of genome instability measured by both the number of CNAs and the genome fraction affected by CNAs (Fig. 1B and D). One cluster is composed of 15 tumors that are characterized by large numbers of whole chromosomes or chromosomal arm amplifications, showing high level of aneuploidy in these tumors. The recurrent CNAs according to GISTIC method were duplications of the entire q arm of 19 chromosome and entire q arm of 20 chromosome. Heterozygous duplications (*n* = 3) and homozygous (*n *= 4) duplications were observed in virtual karyotypes.

The second major cluster of the samples is more heterogeneous, but in general it contains tumors with lower level of genomic instability and contains two subclusters. The first subcluster includes tumors with a low level of aneuploidy (duplication of 1 to 3 chromosomes), samples with focal amplification and deletions of entire chromosomes (mostly chromosomes 1p and 15) and relatively stable samples. It is characterized by recurrent gains of 5p14, 13q21 and losses of 15q and 16q chromosomal arms in line with GISTIC method. Tumors with relatively stable genomes and deletion of chromosome 11 represent a distinctive separate subcluster, with deletion of chromosome 11 and duplications of 5p14, 13q21 identified as recurrent according to GISTIC module.

We determined copy number signature for each of the three tumor clusters (Fig. 1E) and compared it to a known CNA signatures found in human cancers measuring Euclidiean similarity (values ranging 0–1) [30]. The signatures for tumors with low aneuploidy level (both with and without chromosome 11 loss) closely resemble CN1 signature (indicating diploid genome)(Euclidiaen similarity 0.83 and 0.79, respectively). Interestingly, the tumors with high aneuploidy are the most related to CN2 signature (tetraploid genome) (Euclidiaen similarity 0.76) which indicate whole genome doubling (WGD) as an element of the etiology of these sPitNETs (Fig. 1E). Additionally, aneuploidy score for each sPitNET sample was calculated to compare CNAs level in somatotroph tumors and the other common human neoplasms. We observed general relatively low aneuploidy level in sPitNETs (Fig. 1F). However, aneuploidy score of somatotroph tumors with high level of aneuploidy falls in the range of the cancers that are the most affected by genomic instability as uterine carcinoma or testicular germ cell tumors. This analysis also shows that most of the human tumor types including, sPitNETs has bimodal distribution of aneuploidy scores with enrichment of both aneuploidy high and low tumors (Fig. 1F).

#### Verification of the identified DNA copy number changes

**Fig. 2:**
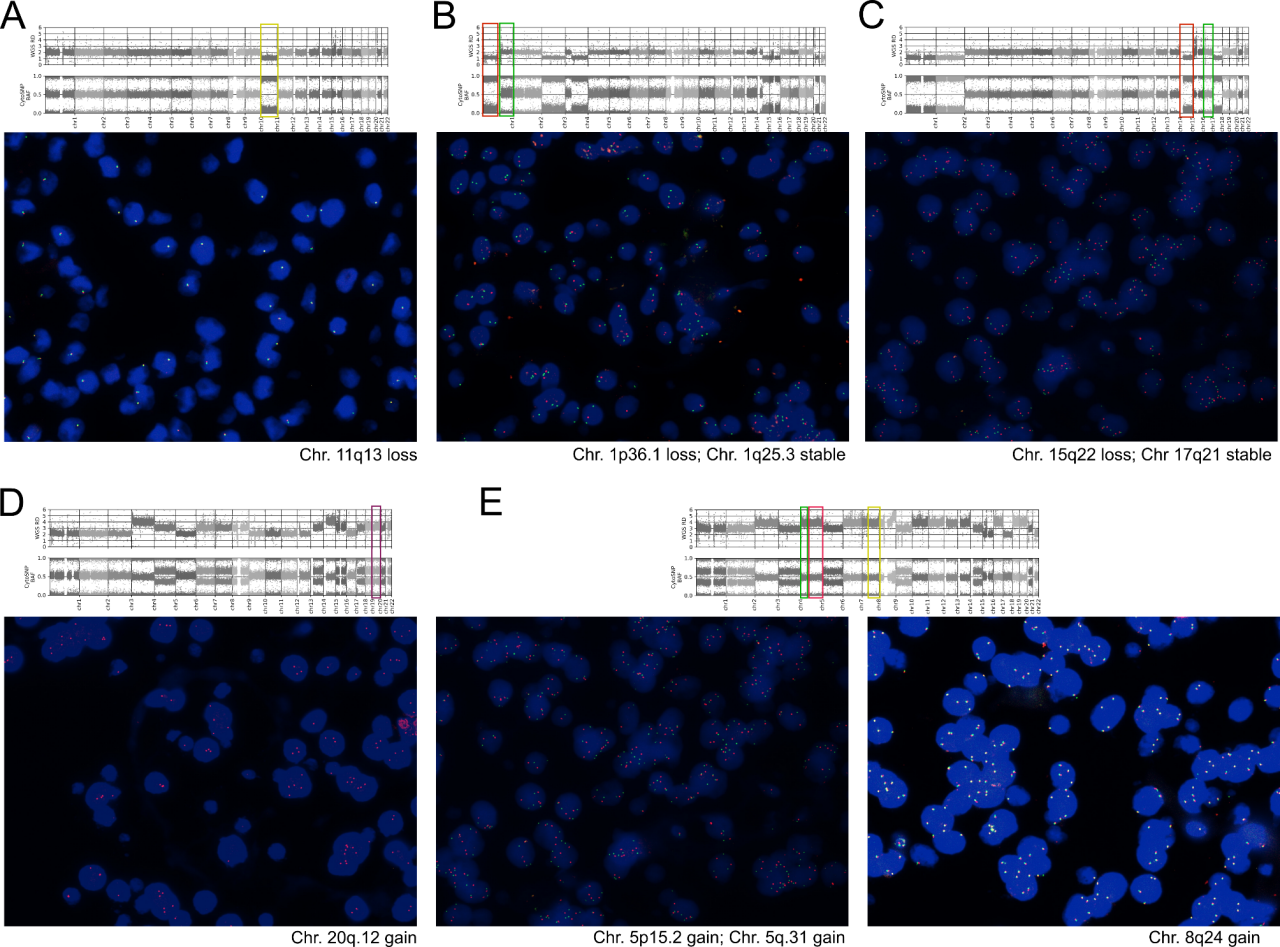
The results of fluorescent in situ hybridization. **A**) The example of chromosome 11 loss; **B** and **C**) The example of the sample with multiple deletions with confirmed chromosome 1p loss (**A**) and 15q22 loss (**B**); **D**) The example of chromosome 20 duplication in sample with multiple chromosomal duplications; **E**) The example of chromosomes 5q15, 5q31 and 8q24 duplication in sample with multiple chromosomal duplications

FISH was applied to validate selected CNAs in tumors with recurrent loss of chromosome 11 (6 cases), multiple chromosomal deletions (3 cases), and large-scale multiple chromosomal duplications (13 cases). In general, CNAs identified with our combined DNA analysis that were covered by FISH probes were confirmed. The presence of additional copies and deletion of one copy detected by FISH corresponded to the gain or loss of the chromosomal arm in the virtual karyotype, respectively. Representative results are presented in Fig. 2, detailed quantification of FISH (% of the cells with CNAs) is presented in the Supplementary Table 1.

In tumors with multiple chromosomal duplications, additional copies of the investigated loci on chromosomes 5, 8, 14, 20 were confirmed in all 13 samples reflecting the chromosome duplication observed in virtual karyotypes. Interestingly, three patterns of chromosomal multiplication were distinguished in this group of FISH cases. In 3/13 tumor samples 3 copies of all examined loci were observed with a percentage of cells with 3 copies of duplicated locus ranging from 38 to 79% in individual tumor samples. The next 3/13 cases had 3 copies of all investigated regions (range from 14 to 83%) but they were also accompanied by 4 copies of these regions in proportion of the cells (range from 13 to 70%). In the remaining 7/13 tumor samples, notably greater intratumor heterogeneity was found in the number of tumor cell clones with multiplicated regions. A proportion of the cells revealed more than 2 additional copies (more than 4 copies) of duplicated chromosomal regions, as exampled in Fig. 2E (details in the Supplementary Table 1).

Chromosome 11 loss was confirmed in each of the six cases that were identified according to genome-wide DNA analysis. The loss of chromosome 11 was observed in more than 80% of the cells, with the exception of 1 sample where it was detected in 27% of the cells. This difference in the content of the cells with chromosome 11 loss clearly corresponds to difference in virtual karyotypes (Sample no.6 in Supplementary Fig. 1).

In the group of multiple chromosomal deletions, the deletion of 1p or chromosome 1 (range from 80 to 85% of cells) and the deletion of 15q (range from 77 to 85% of cells) was observed in all 3 tumor samples.

The accuracy of all probes was tested on selected tumor samples with the most stable genomes according to DNA analysis that served as a negative control. The percentages of deletions (regions of chromosomes 1, 11 and 15) were between 4 and 23% while gains (regions of chromosomes 5, 8, 14, 20) were between 6 and 15%, thus below or at the applied cutoffs as detailed in Supplementary Table 1.

#### Clinical relevance of the cytogenetic profile

**Fig. 3:**
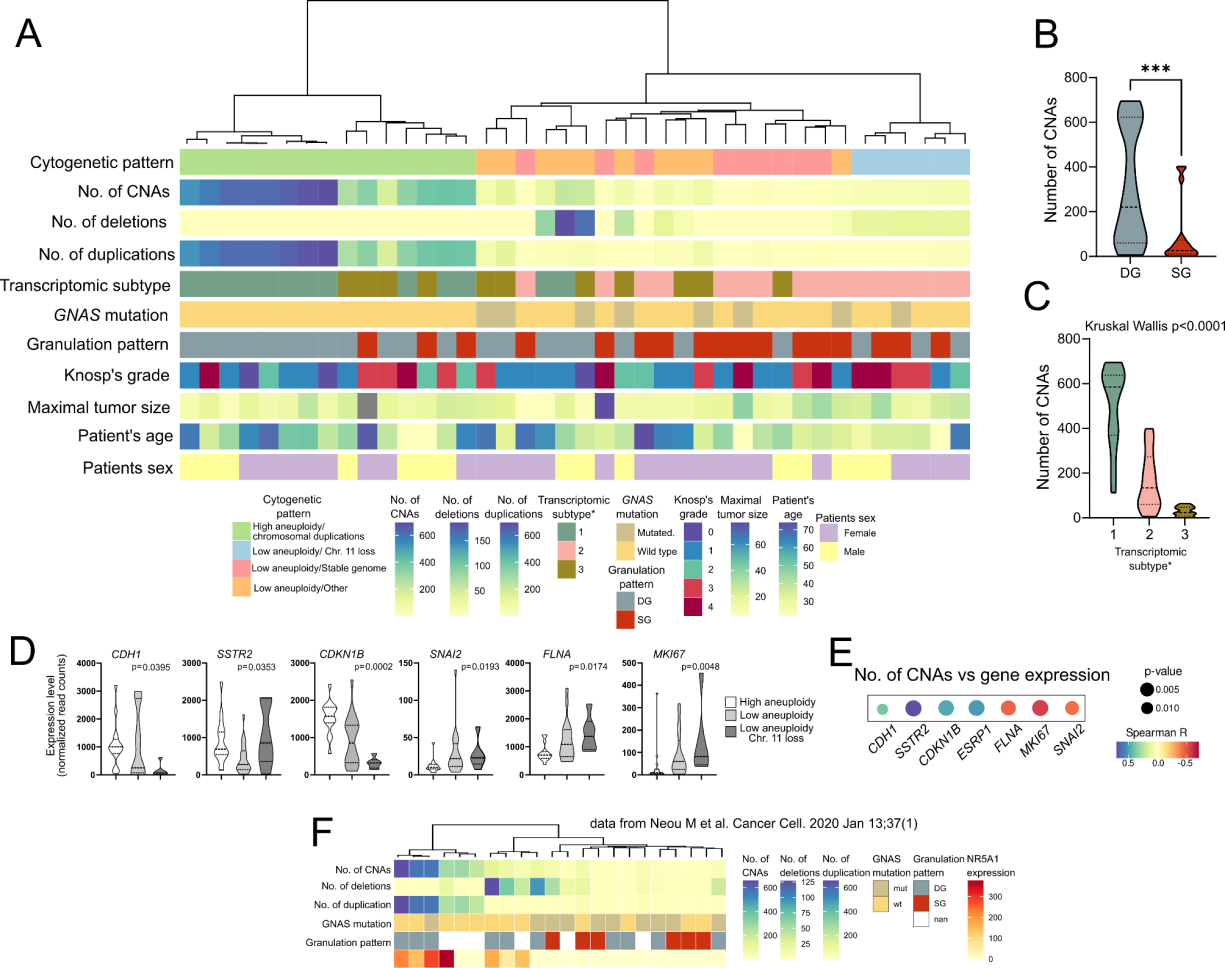
The relationship between CNAs profile and clinico-demographical features. **A**) Summary of the molecular, histological and clinical characteristics. The dendrogram shows the similarity in CNAs pattern; **B**) Comparison of the number of CNAs in densely granulated (DG) and sparsely granulated (SG) sPitNETs, ***- indicate *p* = 0.0001 to 0.001; **C**) Comparison of CNAs number in tumors classified into one three transcriptomic subtypes according to previous profiling [20] Subtype 1 are double positive PIT1/SF1 positive DG tumors, Subtype 2 are PIT1 positive, mostly DG tumors with common GNAS mutations, Subtype 3 are mostly SG tumors; **D**) The expression of known prognosis-related genes in sPitNETs stratified according to CNAs based hierarchical clustering into: high aneuploidy, low aneuploidy and low aneuploidy/ chromosome 11 loss tumors, p-value from Kruskal-Wallis test; **D**) The correlation between aneuploidy level (CNAs number) in somatotroph tumor and the expression of known prognosis-related genes; **E**) Results of the analysis of independent validation set, sPitNETs from previous study [8]

We observed the relationship between selected tumor histological characteristics and cytogenetic profile as presented in Fig. 3A. Comparison of two clusters of tumor samples grouped according to cytogenetic profile showed that the cluster of highly aneuploid tumors contains only *GNAS* wild type and is mainly dominated by DG somatotroph tumors. The differences in the proportions of *GNAS*-mutated and DG tumors were significant (*p* = 0.0162 and *p* = 0.0464, respectively). Higher chromosomal instability was observed in DG than in SG tumors (Fig. 3B). No difference was observed between invasive and noninvasive tumors. We did not observe the relation between CNA status and other clinical/demographical features including patient’s age at surgery and sex, GH1 and IGF-1 in patients, as well as tumor size and invasive growth status.

In our previous study, based on tumor samples that we used here for copy number analysis, we found clearly distinct transcriptomic subtypes of somatotroph pituitary tumors [20]. The relevance of this subclassification of somatotroph PitNETs was demonstrated by independent research [48]. In this study, we observe the relation between transcriptomic and cytogenetic classification. The cluster of highly aneuploid samples contains nearly all Subtype 1 tumors that are positive for *NR5A1* (SF-1) expression, while only two *NR5A1* positive tumors were found in a cluster of more stable tumors. Each of these 2 samples were affected by multiple chromosomal deletions, including chromosomes 1p, 15q, 16 and 22. Samples representing transcriptomic Subtype 3 were found exclusively in the cluster of tumors with lower instability. All the tumors with chromosome 11 deletion were transcriptomic Subtype 3 tumors. The clusters did not differ in the proportion of transcriptomic Subtype 2 tumors. These data are shown in Fig. 3C. A clear difference in the genomic instability measured with the number of CNAs was observed between the transcriptomic subtypes of the PitNETs. It was the highest in transcriptomic Subtype 1 (*NR5A1*/SF-1 positive tumors), a moderate level was observed in Subtype 2 samples while Subtype 3 samples were the most stable (Fig. 3C).

Tumor expression of particular genes was found of prognostic/predictive value in acromegaly patients, These genes include basically *CDH1* [37, 38] and *SSTR2* [39, 40] but also *CDKN1B* [37] *SNAI2* [41], *FLNA* [42], *ARRB1* [43, 44], *SNAI1* [45] *RORC* [45, 46] *ESRP1* [38], and *MKI67* [46]. Using RNAseq data, we compared the expression levels of these genes in tumors with high and low level aneuploidy sPitNETs, distinguishing tumors with chromosome 11 loss. Different expression of *CDH1*, *SSTR2*, *CDKN1B*, *ESRP1*, *SNAI2*, *FLNA* and *MKI67* was found and the expression levels of these genes are correlated with CNAs number (Fig. 3D, E). This result indicate more favorable genes expression profile (higher *CDH1*, *SSTR2*, *CDKN1B*, *ESRP1* and lower *SNAI2*, *FLNA*, and *MKI67*) as related to higher aneuploidy level in sPitNETs.

We used an independent data from previously investigated sPitNETs [8] to validate our observations. Cohesively with our methodology DNA copy number profiling by Neou at al. was based on whole genome SNP arrays and analysis included both BAF and signal intensity [8]. In general, the clustering of the 22 independent sPitNETs showed the CNAs patterns reflecting the one observed in our samples: two major cluster including one with high aneuploidy level due to large scale chromosomal duplications and the other with chromosomal deletions or relatively stable genomes (Fig. 3F, details in Supplementary Fig. 2A). Unlike in our study, only one patient with chromosome 11 deletion was found in the validation dataset. According to our observations, cluster of highly aneuploid samples in validation group contained most of *NR5A1*-expressing tumors, mainly GNAS wild type and densely granulated sPitNETs, while low aneuploidy tumors included most of GNAS mutated tumors and were enriched in SG ones (Fig. 3F). As in our results signature of highly aneuploid sPitNETs were the most similar to CN2, while low aneuploidy tumors to CN1 pan-cancer signature (Supplementary Fig. 2B).

### Functional role of CNAs and genomic instability in somatotroph PitNETs

#### Influence of copy number change on transcriptome

**Fig. 4:**
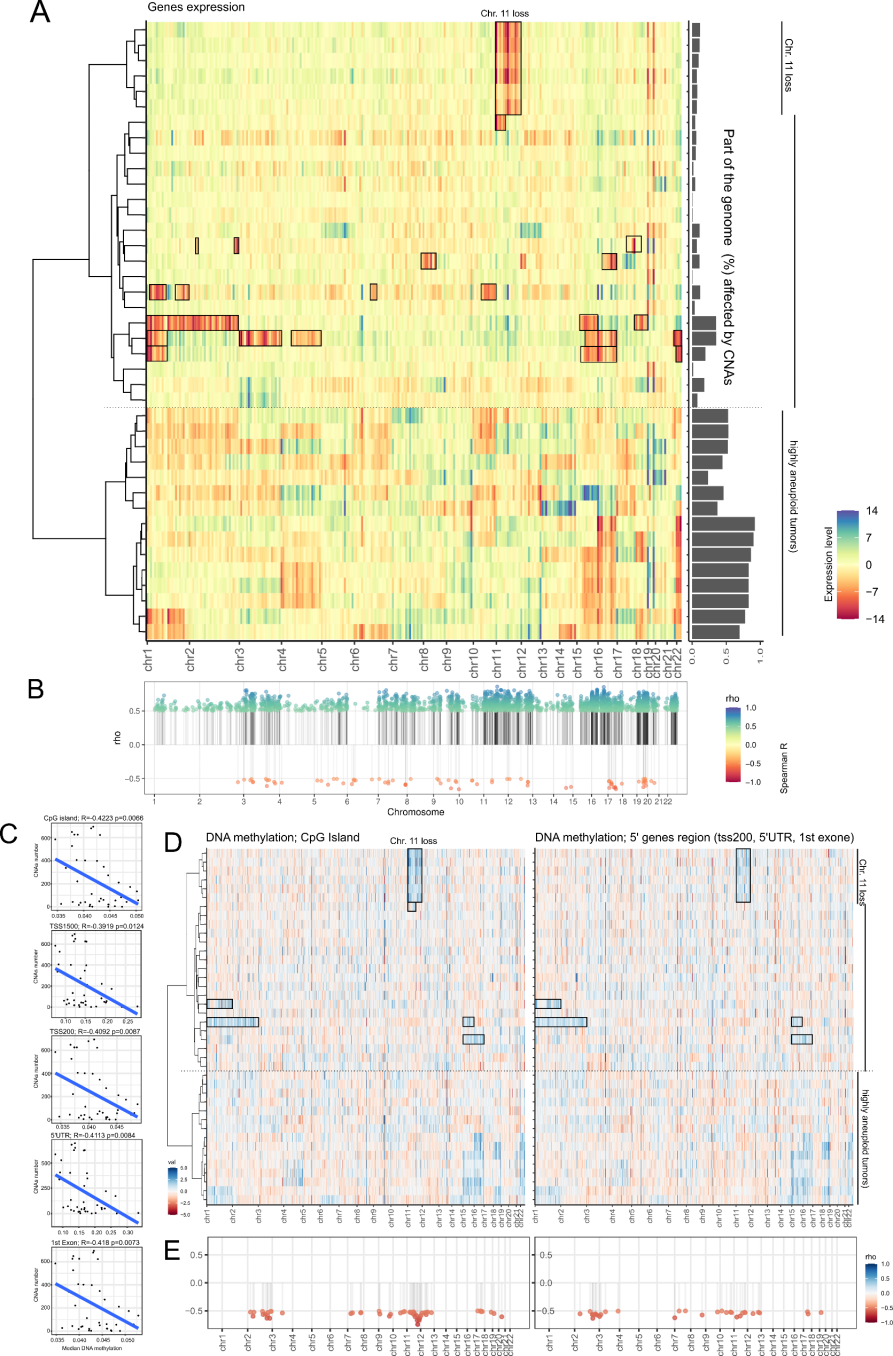
Relationship between CNAs and genes expression or DNA methylation profiles. **A**) Genes expression in tumor samples clustered according to cytogenetic pattern (reflecting Fig. 1B). The heat map shows the mean expression of genes encoded by each chromosome band. The boxes indicate sites of chromosomal loss. The dendrogram shows similarity in the pattern of CNAs; **B**) Correlation between the expression and copy number value of each gene, Spearman *R *> |0.5| was used as a cut-off point for moderate/high correlation. Each dot represents a particular gene; **C**) Correlation between CNAs count and median DNA methylation (median β-value) at specified category of CpG sites; **D**) Normalized, scaled DNA methylation level in individual sPitNETs showing difference in DNA methylation at CpG island and genes promoters at deleted chromosomal regions from the methylation level from the other chromosomal regions. Boxes indicate sites of chromosomal loss. The dendrograms show similarity in pattern of CNAs; **E**) Correlation between expression and copy number value of each gene, Pearson *R *> |0.5| was used as a cut-off point for moderate/high correlation. Each dot represents a chromosomal band

RNA sequencing was performed for the 40 samples included in the cytogenetic profiling. To investigate the functional consequences of the cytogenetic changes, the expression levels of genes across the chromosomal bands were examined. In the cluster of tumors with a low level of aneuploidy, we clearly observed that chromosomal deletions resulted in the notable decrease in the expression of genes encoded by the deleted regions. Recurrent deletions of chromosome 11 were reflected in downregulation of the genes located in chromosome 11. The expression of genes located in deleted chromosomal regions is clearly decreased in samples affected by multiple deletions. The relationship between copy number gain and increased expression of the genes encoded by duplicated regions was also found in the cluster of low aneuploidy level tumors. Duplications of chromosomes 3, 5, 8, 12, 17, 18 and 20 found in individual samples were clearly reflected by a higher expression of genes encoded by these chromosomal regions. Distinct relationship between cytogenetic and transcriptomic profiles was observed in tumors that are affected by multiple chromosomal duplications but not deletions. In these patients, decreased genes expression was found in genomic regions with normal DNA copy number, while increased chromosome copy number was related to increased expression of corresponding genes. Genome-wide expression profiles are presented in Fig. 4A.

For an alternative approach to investigatethe influence of DNA copy number on gene expression for each protein coding gene, we determined the correlation between the expression level of each protein coding gene and its DNA copy number level. As a result, we observed a significant moderate and high correlation (Spearman *R* > 0.5) in 2,490 genes, including 2413 genes with positive and only 77 genes with negative correlation, confirming the role of CNAs in genes expression (Fig. 4B).

#### Relationship between copy number and DNA methylation profile

A correlation between DNA methylation (estimated as the overall β-value of DNA microarrays) and number of CNAs for pituitary tumors was previously reported [8] but such general relationship was not observed in our data. In turn, such DNA methylation/CNA correlation was found for the specific categories of CpG sites stratified according to CpG island (island, shelf, shore and open sea) or gene (tss1500, tss200, 5’UTR, 1st Exon, gene body, 3’ UTR, intergenic) (Fig. 4C). We observed an inverse correlation between the number of CNAs and median DNA methylation on CpG island as well as tss200, 5’UTR and 1st Exon (that can be collectively recognized as genes’ 5’ regions) in the regions that are characterized by low DNA methylation (as shown in the Supplementary Fig. 3).

When DNA methylation/CNA correlation for either CpG island or genes 5’ regions was calculated for each chromosomal band independently we observed that moderate/high correlation was present mostly in the chromosomal regions that undergo deletions in sPitNETs, especially in case of chromosome 11 loss (Fig. 4D and E). This indicates that chromosomal loss tends to be associated with hypermethylation of CpG islands and 5’ gene regions in the remaining chromosome.

#### Genes expression in tumors with deletions of chromosome 11 and copy number stable somatotroph PitNETs

**Fig. 5:**
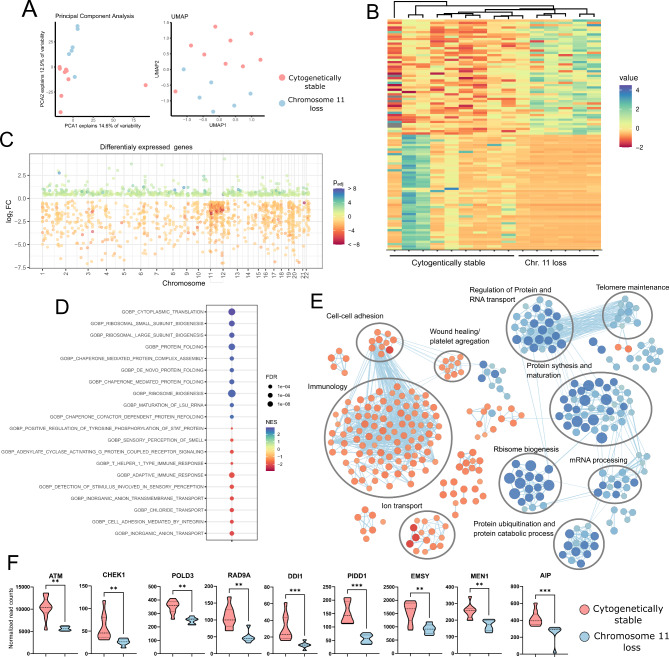
Comparison of gene expression between sPitNETs with chromosome 11 deletion and cytogenetically stable ones. **A**) Results of the dimensionality reduction analysis; **B**) Clustering and the expression of the most variably expressed genes; **C**) Differentially expressed genes plotted according to chromosomal location. Each dot represents a particular gene. **D**) Top 10 most enriched upregulated and downregulated GO biological processes; **E**) All significantly enriched GO biological processes classified according to a common physiological role; **F**) Comparison of the expression of known cancer-related genes encoded on chromosome 11 identified as differentially expressed. **- indicate *p* = 0.001 to 0.01, ***- indicate *p* = 0.0001 to 0.001

Chromosome 11 deletions are the most common recurrent cytogenetic change that was found in tumors with relatively stable genomes. Complete deletion of chromosome 11 was observed in 6 tumor samples, while deletion of only 11p was observed in one tumor. To investigate the relevance of chromosome 11 loss we compared genes expression in 6 samples harboring this CNA with the set of samples with the most stable genomes (the lowest number of CNAs, *n* = 9). Both the dimensionality reduction methods and the hierarchical clustering indicate these tumor groups are distinctive in the whole transcriptomic profile (Fig. 5A and B). We identified 928 DEGs with |FC|>2 that were presented in the Supplementary Table 2. When we looked for the distribution of DEGs at the corresponding chromosomal arms, we observed that tumors with loss of chr. 11 show a significant downregulation of genes located at chromosome 11 indicating a direct consequence of chromosome deletion. However, a number of DEGs that are encoded genome-wide was also observed (Fig. 5C).

Identifying a high number of DEGs, we focused on the GSEA to identify the processes related to chromosome 11 loss. Using the GO Biological Processes database, we identified 342 processes that were significantly enriched (Supplementary Table 3). The top 20 enriched processes are presented in Fig. 5D. Most of the terms were directly related to a few specific categories of biological processes. Tumors with deletion of chromosome 11 were characterized by upregulation of processes related to protein synthesis and maturation, ribosome biogenesis, polyubiquitin-related protein degradation, and biomolecule transport that probably represent a compensatory mechanism for the change in expression at protein level. In general, such a compensatory mechanism in human tumors with genomic instability has been previously described [47]. Interestingly, DNA copy number stable tumors were characterized by upregulation of the process related to immune response and ion transport. Results are presented in Fig. 5E.

To determine the possible role of chromosome 11 deletion as a tumor driver, we focused on the expression of genes encoded on this chromosome that are downregulated in samples with chr. 11 loss. Comparison of samples with chromosome 11 loss with copy number stable samples indicated 411 such a genes (Supplementary Table 2) including the well-known tumor suppressor *MEN-1*, important DNA damage checkpoint genes *ATM* and *CHEK1* as well as the other genes involved in DNA repair *POLD4*,* POLD3*,* RAD9A*,* DDI1*,* PIDD1 and EMSY* (Fig. 5F). Furthermore, manual data inspection showed downregulation of *AIP* gene in tumors with loss of chromosome (this difference did not cross the significance threshold in comparison of the whole transcriptome with DESeq2).

#### Difference in gene expression in highly aneuploid and copy number stable tumors

**Fig. 6:**
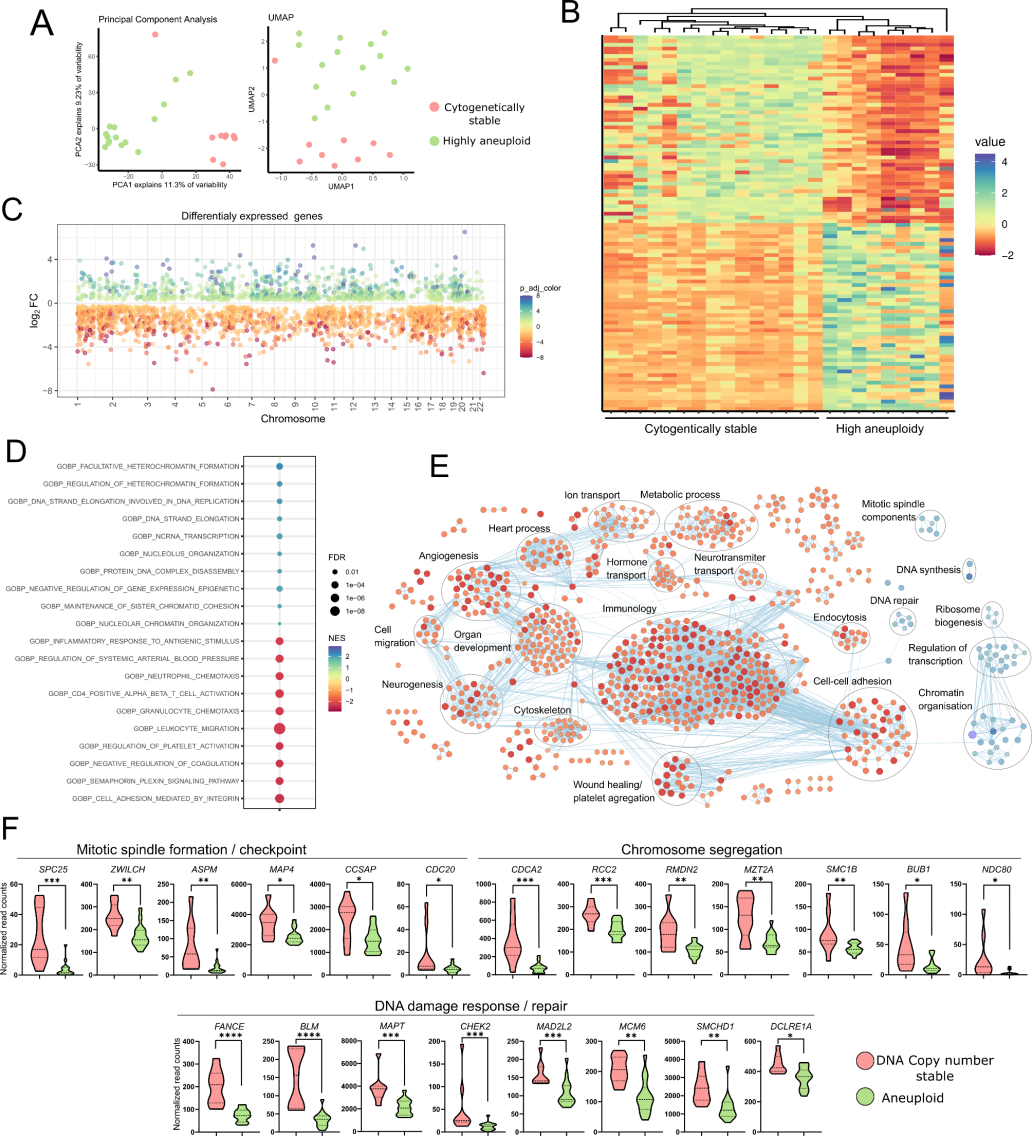
Comparison of gene expression in sPitNETs with a high level of aneuploidy (multiple chromosomal duplications) and cytogenetically stable tumors. **A**) Results of the dimensionality reduction analysis results; **B**) Whole transcriptome-based clustering and the expression of top the most variably expressed genes; **C**) Differentially expressed genes plotted according to chromosomal location. Each dot represents a particular gene. **D**) Top 10 most enriched upregulated and downregulated GO biological processes; **E**) All significantly enriched GO biological processes classified according to a common physiological role; **F**) Comparison of the expression of mitotic spindle-, chromosome segregation-, DNA damage response-related genes that were identified as downregulated in tumors with high aneuploidy (multiple chromosomal duplications). *- indicates *p* = 0.01 to 0.05, **- indicates *p* = 0.001 to 0.01, ***- indicates *p* = 0.0001 to 0.001, ****- indicates *p* > 0.0001

Genes expression in copy number stable tumors and tumors with high level of aneuploidy (all the tumors of aneuploidy high cluster, *n* = 15) was also compared (Fig. 6A). As a result, we identified 2049 DEGs with |FC|>2 that included mainly downregulated genes in aneuploid samples (1516/2049 DEGs), as shown in Fig. 6B, C and listed in the Supplementary Table 4. In aneuploid tumors, we did not observe unequivocal upregulation of the genes located at commonly gained chromosomes such as chr. 5, 8, 9, 14, or 20 that could be expected as a consequence of chromosomal duplication. In turn, we rather observe that upregulated and downregulated genes are evenly distributed throughout the genome irrespective of CNA status, indicating both direct and indirect deregulation of genes expression (Fig. 6C). Therefore, the high level of aneuploidy is related to whole transcriptome change rather than local expression dysregulation that was observed in tumors with chr. 11 loss.

GSEA with GO Biological Processes indicated 867 significantly enriched processes (the top 10 enriched processes are presented in Fig. 6D, listed in details in Supplementary Table 5). The processes enriched the most in aneuploid tumors were related to specific categories of pathways related to regulation of mitotic spindle formation, DNA synthesis, DNA repair, chromatin organization, regulation of transcription and ribosome biogenesis. In turn, the processes most enriched in cytogenetically stable tumors were those related to immune response, cell-cell adhesion, angiogenesis, wound healing/platelet aggregation, neurogenesis, ion transport, as well as hormone and neurotransmitter transport (Fig. 6E).

For the search of the possible causative feature of aneuploidy, we looked at the genes downregulated in aneuploid tumors that are known to be related to the process of organizing the genetic material during cell division and DNA repair. We found that few know relevant genes related to genome stability that are significantly downregulated in aneuploid tumors, including the DNA damage response and repair genes *FNACF*, *BLM*, *MAPT*, *CHEK2*, *MAD2L2*, *MCM6*, *SMCHD1* and *DCLRE1A*; mitotic spindle formation and checkpoint genes *SPC25*, *ZWILCH*, *ASPM*, *MAP4*, *CCSAP*, *CDC20*; genes involved in chromosome segregation *CDCA2*, *RCC2*, *RMDN2*, *MZT2A*, *SMC1B*, *BUB1* and *NDC80* (Fig. 6F). Due to the specific function, downregulation of these genes possibly contribute to genome instability in aneuploid tumors.

#### Tumor microenvironment and cytogenetic status in somatotroph PitNETs

**Fig. 7:**
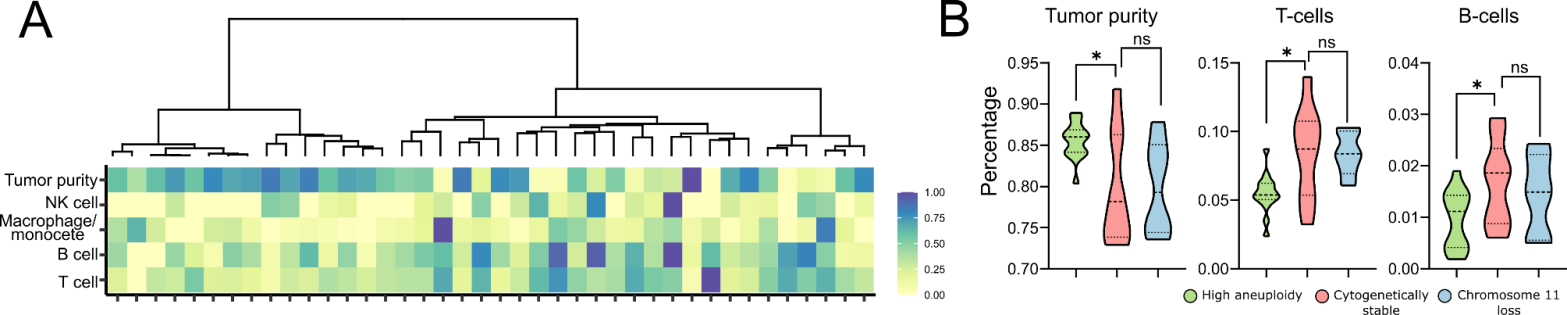
The content of immune cells and tumor purity based on deconvolution of DNA methylation data (EPIC human methylation arrays, Illumina). **A**) Normalized immune cell content in the entire group of sPitNETs. Dendrogram shows similarity in the pattern of CNAs; **B**) Comparison of estimated tumor purity, as well as T-cells and B-cell content, in sPitNETs with high aneuploidy (multiple chromosomal duplications), chr. 11 loss, and cytogenetically stable tumors. * indicate *p* = 0.01 to 0.05

As mentioned above, both comparisons of genes expression showed the increased expression of immune response genes in cytogenetically stable tumors compared to tumors with chr. 11 loss and multiple duplications, as reflected by a high number of the enriched immune-related GO biological processes in stable tumors. For further investigation of the relationship between the tumor microenvironment and CNAs profile, we applied deconvolution method based on methylation of genome-wide DNA data to estimate the immune cell content in tumor samples (Fig. 7A). The results were used to compare highly aneuploid tumors and tumors with loss of chromosome 11 with cytogenetically stable sPitNETs. This confirmed a significantly lower rate of T cells and B-cells as well as higher tumor purity in aneuploid sPitNET, but no significant difference was observed between tumors with chr. 11 loss and those cytogenetically stable (Fig. 7B); however, a relatively low immune cell fraction was calculated with a median < 10% for T cells and < 5% for B-cell in each group.

## Discussion

Pituitary tumors are characterized by a low incidence of point mutations with single amino acid substitutions as the only highly recurrent, while somatic changes in the DNA copy number changes were commonly detected in somatotroph tumors with various methodological approaches [7–18]. The available results indicate a wide spectrum of cytogenetic alterations of PitNETs raging from relatively stable genomes to genome-wide large scale chromosomal abnormalities, as also observed in our study. The results of particular studies differ in the reported copy number profiles of somatotroph PitNETs.

In our study, we found that multiple chromosomal duplications (affecting more than 50% of the genome) and chromosome 11 loss with the rest of genome intact are two of the most common recurrent cytogenetic abnormality patterns in sPitNETs. The cases of somatotroph tumors with multiple chromosomal duplications were identified in some of the studies that applied a genome-wide methodology [8, 10, 48] but were not reported in many others [11, 15, 49]. Importantly, none of the previous studies showed as high prevalence of this genome-wide chromosomes duplication in sPitNETs as found in our research. Similarly, somatotroph tumors with chromosome 11 loss were reported by some of the authors [10, 48] but they were not observed by the others [11].

Considering the discrepancy between the former results, we applied two independent, genome-wide methods for identification of copy number abnormalities. Our CNAs results were inferred based on three independent information layers: the relative sequencing read depth from WGS, the level of SNP microarray probe signal, and allelic distribution at SNPs. Based on our experience [50], we suspect that incidence of genome-wide multiple chromosomal duplications in pituitary tumors could be underestimated in previous studies, especially those that were based on the tools that do not include analysis of SNPs biallelic frequency into account. SNPs distribution analysis is not included when inferring CNAs from a methylation microarray or low-pass WGS. We are aware that the information from SNPs is crucial for establishing the normal copy number level in highly aneuploid samples, where the *n* = 2 level is commonly improperly set by default by analytical algorithms [50]. Thus, we are convinced that combining data from independent platforms to generate a comprehensive virtual karyotype increases the reliability of the results. To provide an independent, microscopic validation of the identified CNAs, we used FISH with probes located at the most frequently affected chromosomal regions. Visual microscopic inspection and quantification of the fluorescent signal confirmed the observation of virtual karyotyping. Furthermore, microscopic observations show that a group of highly aneuploid tumors is characterized by notable intertumoral heterogeneity. The majority of these sPitNETs contained tumor cells with one additional copy of the examined chromosomal region, but also a notable proportion of the cells with various higher copy numbers of these loci, indicating chromosomal instability. This observation clearly contradicts the former common opinion that pituitary tumors are nearly monoclonal and supports the concept of their polyclonal evolution [51].

An unsupervised clustering based on the similarity of the CNAs pattern showed two main clusters of the somatotroph PitNETs. First cluster included tumors with multiple chromosomal duplications but without chromosomal losses. Second cluster was more heterogeneous, it included tumors with multiple deletions, those with relatively stable genomes, as well as sPitNETs with chromosome 11 deletion distinguished as a clear separate subclusters. In our study, the relationship between some clinicopathological characteristics was observed showing that genomic instability is higher in densely granulated sPitNETs, as well as confirming the previously reported high level of CNA in *GNAS* wild type tumors [16, 49], which is consistent with different proportion of *GNAS*-mutated tumors in the main cytogenetic clusters of sPitNETs.

Very recently, a clear relationship between loss of heterozygosity and aggressiveness of pituitary tumors was reported [52]. Aggressive, recurrent PitNETs were characterized by high level of LOH, resulting basically from chromosomal deletions, however limited number of sPitNETs was included in this study [52]. The relationship between prognosis and increased CNAs (both deletions and gains) was also found in lactotroph PitNETs [10]. We were not able to analyze the prognostic role of cytogenetic profiles in our cohort due to a relatively low follow-up time and lack of aggressive refractory tumors in study group. However, when we considered the status of granulation pattern as well as the expression of known prognosis related genes [35–44] we found that in somatotroph tumors favorable features are related to high aneuploidy and multiple chromosomal gains. Dense granulation, higher expression of *CDH1* (E cadherin), *SSTR2*, *CDKN1B* (P27) and lower expression of *SNAI2* and *MKI67* were related to higher number of CNAs and chromosomal duplications indicating that highly aneuploid tumors are potentially of better prognosis than more stable sPitNETs, especially those with chromosome 11 loss which showed the least favorable expression pattern.

Results from previous molecular profiling of GH-secreting pituitary tumors showed their 3, clinically relevant molecular subtypes: DG PIT1/SF1, DG PIT1, and SG PIT1 tumors [20, 48]. All tumor samples included in our study have previously been classified into molecular subtypes based on transcriptomic data [20] and with current work we compared the subtypes in terms of the incidence of copy number profile. This shows that double positive PIT1/SF-1 DG tumors are highly affected by large-scale genome instability and 11/13 of these tumors are affected by a high level of aneuploidy while the remaining 2 harbor multiple deletions. PIT1/SF-1 positive somatotroph tumors have been occasionally reported through the years [8, 53–59] and recently they were characterized as a specific subtype [20, 48]. This subgroup of sPitNETs is composed of wild-type *GNAS* tumors that express GIPR receptor (the receptor that is considered related to paradoxical GH response after oral glucose load [20]). Consequently, *GIPR* expression has previously been shown to be related to higher CNAs level [16]. In turn, the most common deletion found in our study group, loss of chromosome 11, was identified in tumors – with relatively stable genomes, wild-type *GNAS*, which all belong to a particular transcriptomic subtype of tumors according to our previous transcriptomic subclassification [20]. Chromosome 11 deletions were previously observed in sPitNET [10, 48]. They were also recently identified as a hallmark of SG PIT1 somatotroph tumors classified by Dottermusch M et al. [48] which is consistent with our observation that chromosome 11 loss is only present in tumors from our transcriptomic Subtype 3.

In general, the role of chromosomal imbalance in cancer is complex. It may have a tumor-promoting effect and provide a selective advantage in clonal tumor evolution, but it also may be neutral or even tumor suppressive, but tolerated in neoplastic cells due to defects in checkpoint mechanisms [60–62]. In our study, we examined the possible functional relevance of CNAs by analyzing gene density in the cytogenetically affected regions, comparing the CNAs pattern with the transcriptomic profile in the individual tumor samples, and analyzing the correlation between copy number and genes expression by comparing transcriptomes between the groups of tumors with a recurrent cytogenetic patterns with cytogenetically stable tumors. We found that chromosomal duplications are significantly more common than chromosomal losses, as previously observed [10]. Our results indicate that these two classes of CNAs have distinct roles in sPitNETs. Chromosomal gains (specifically those most common in the entire group) were found in regions with low gene density, while the deletions were found in gene dense regions including chromosome 11, which is among chromosomes with the highest gene content [63]. We also found that deletions were directly related to decreased expression of the affected genes indicating chromosomal deletions are more likely to be tumor-promoting by local gene expression than chromosomal duplications that occur on a much larger scale and are associated with a whole transcriptome dysregulation.

When we compared the copy number signatures for each group of sPitNETs with the reference pan-cancer signatures [30] it appeared that copy number pattern in highly aneuploid sPitNETs closely relate to CN2 signature of tetraploid genome. It indicates the role of WGD in etiology of this specific group of sPitNETs. Genome doubling is the phenomenon observed ~ 30% of human cancer that drives oncogenesis through increasing chromosomal instability and changing spatial chromatin organization [64, 65]. We conclude there is a substantial difference in the etiology of highly aneuploid sPitNETs that develop thorough WGD and the sPitNETs with low aneuploidy level, especially with those harboring where chromosome 11 loss.

When we compared genes expression in highly aneuploid sPitNETs with cytogenetically stable ones, we found that genes upregulated in aneuploid tumors were mainly related to DNA repair, regulation of the mitotic spindle, chromatin organization, and regulation of transcription, which can be considered as a trace of cellular response to chromosomal abnormalities and putative WGD as oncogenic driver. Duplications of the particular chromosomes were not related to upregulation of the corresponding genes, as generally observed in WGD in cancer [66]. Amplification of chromosome 20 was previously found to be related to increased *GNAS* expression [16] or considered as a probable indirect cause of *GNAS* upregulation [7]. In our study the duplication of chromosome 20 was found and confirmed in all tumors with multiple chromosomal duplications, but we did not find a relation between chromosome 20 gains and the increase in *GNAS* expression. We did not observe a general correlation between *GNAS* expression and the copy number of the *GNAS* locus, as well as a difference in *GNAS* mRNA level in cytogenetically stable and aneuploid tumors and copy number.

Chromosome 11 loss can be considered as tumor driving due to its recurrent nature and clear effect on genes expression. Comparing tumors with chromosome 11 loss and cytogenetically stable tumors confirm the direct downregulation of the genes in this chromosome, including important tumorigenesis related genes such as *ATM*, *CHEK1*, and *EMSY* that are involved in DNA repair, as well as the pituitary tumor-related genes *MEN-1* and *AIP* (both at chromosome 11.q13). Loss of function mutations or deletions of these two genes are observed in the familial form of acromegaly as a manifestation of familial isolated pituitary adenoma and multiple endocrine neoplasia type 1 syndromes [67]. In general, *AIP* or *MEN1* point mutations in sporadic sPitNET are very rare [67] and previous studies on recurrent 11q13 deletion, raising the suggestion that MEN1 downregulation have tumorigenic role [68, 69]. Chromosome 11 loss was recently described as the second hit inactivating *MEN1* in a case of sporadic sPitNET [70]. In our study, a clear decrease in *MEN1* and *AIP* was observed in sporadic sPitNETs with loss of chromosome 11, indicating downregulation of these two genes as probably tumorigenic.

A relationship between DNA methylation and the number of CNAs in pituitary tumors has been previously reported [8]. No such general correlation was observed in our study, but we found that there is a negative correlation between DNA methylation on the CpG island and gene promoters and DNA copy number. Importantly, a high level of methylation in these regions is considered to be related to transcriptional inactivity of genes [71]. The relationship between methylation and copy number was more clear for chromosomal deletion. In samples with chromosomal loss, we noticed a DNA hypermethylation of the remaining chromosome. We consider this as a kind of addictive mechanism contributing to downregulation of gene expression at particular chromosomal regions. Our data indicate that this phenomenon was the most pronounced in the case of recurrent deletions of chromosome 11. [7, 16, 66–70]

One of the important issues on the role of chromosomal imbalance in cancer biology is the relationship between genomic instability and immune response in cancer. In general, lower immune infiltration was observed in tumors with high chromosome imbalance in human cancers [72, 73]. However, the mechanistic relationship between genomic instability and the immune system is more complex [74, 75]. In our study, the comparisons of gene expression between highly aneuploid and chromosome 11 deleted sPitNETs with cytogenetically stable tumors showed the expression pattern indicating a higher immune component in tumors with stable genomes. Consequently, when we estimated the content of immune cells using deconvolution of DNA methylation data, we detected a lower proportion of T and B-cells in group of highly aneuploid tumors compared to cytogenetically stable tumors (similar difference between tumors with chr. 11 loss and stable tumors did not cross the significance threshold). Thus, these preliminary data suggest a similar relationship between CNAs level and microenvironment composition as in other human cancers; however, it requires more detailed verification. In fact, some variability in the immune component in sPitNETs was previously observed and was shown to be related to pharmacological treatment outcome [35, 36]. However, the content of immune cells in pituitary tumor is generally low [76] and our personal experience also indicates that clear immune infiltration in PitNETs is uncommon.

Importantly, the incidence of large-scale genomic instability in a cancer can be used in synthetic lethality therapeutic approach targeting aneuploidy. Inhibition of the spindle assembly checkpoint has been found to selectively eliminate aneuploid cancer cells by inducing further chromosomal imbalance, breaking the barrier tolerance of cells to the aneuploidy level, and enhancing their level of chromosomal instability [77, 78]. The high level of aneuploidy in somatotropic tumors allows for the consideration of this therapeutic approach in some patients with acromegaly in whom CNA status could serve as a potential biomarker.

## Conclusions

In our study, we presented a complete and reliable catalog of CNAs in sPitNETs, based on genome-wide DNA analysis methods and FISH. Based on unsupervised clustering, we distinguished 3 cytogenetic groups of these tumors: highly aneuploid sPitNETs, low aneuploidy tumors and the low aneuploidy tumors with chromosome 11 loss. Highly aneuploid tumors are *GNAS* wild type tumors that are mostly DG sPitNETs expressing *NR5A1* (SF-1) with the favorable expression of prognosis-related genes including high *CDH1*, *CDKN1B* and *SSTR2* levels. In turn, tumors with low aneuploidy are enriched in unfavorable histology of SG pattern and has higher immune infiltration as observed in data deconvolution.

Our results show for the first time the massive extent of aneuploidy in somatotroph pituitary tumors– incidence of multiple chromosomal duplications with intratumoral cytogenetic heterogeneity as a very common feature of sPitNETs. The cytogenetic profile in these tumors resemble CN2 pan-cancer signature indicating the role of WGD in their pathogenesis. We also documented the role of previously observed recurrent chromosome 11 loss. In opposite to chromosomal gains, genomic deletions occur mainly in gene-rich genomic regions and has clear impact on decreased genes expression including the role of chromosome 11 deletion on downregulation of known pituitary tumorigenesis-related genes as *MEN1* and *AIP*.

## Electronic supplementary material

Below is the link to the electronic supplementary material.

Supplementary Material 1

Supplementary Material 2

## Data Availability

EPIC array data is available at Gene Expression Omnibus: GSE226764; RNA-Seq data is available at ArrayExpress: E-MTAB-11889. Further enquiries can be directed to the corresponding author.

## Abbreviations

BAF: B allele frequency
CNAs: Copy number abnormalities
DEGs: Differentially expressed genes
DG: Densely granulated
FC: Fold Change
FFPE: Formalin-fixed paraffin-embedded
FISH: Fluorescence hybridization in situ
GH: Growth hormone
GIPR: Gastric inhibitory polypeptide receptor
GO: Gene ontology
GSEA: Gene set enrichment analysis
IGF-1: Insulin-like growth factor-1
OGTT: Oral Glucose Tolerance Test
PitNET: Pituitary neuroendocrine tumor
qRT-PCR: Quantitative reverse transcription PCR
RNAseq: RNA sequencing
SG: Sparsely granulated
SNP: Single nucleotide polymorphism
SRLs: Somatostatin receptors ligands
TSH: Thyroid-stimulating hormone
UTR: Untranslated region
WGS: Whole genome sequencing
