# Supplementary figures and images for: High level of aneuploidy and recurrent loss of chromosome 11 as relevant features of somatotroph pituitary tumors
Source: J Transl Med. 2024 Nov 4;22:994. doi: 10.1186/s12967-024-05736-0 (PMC11536836; doi:10.1186/s12967-024-05736-0)

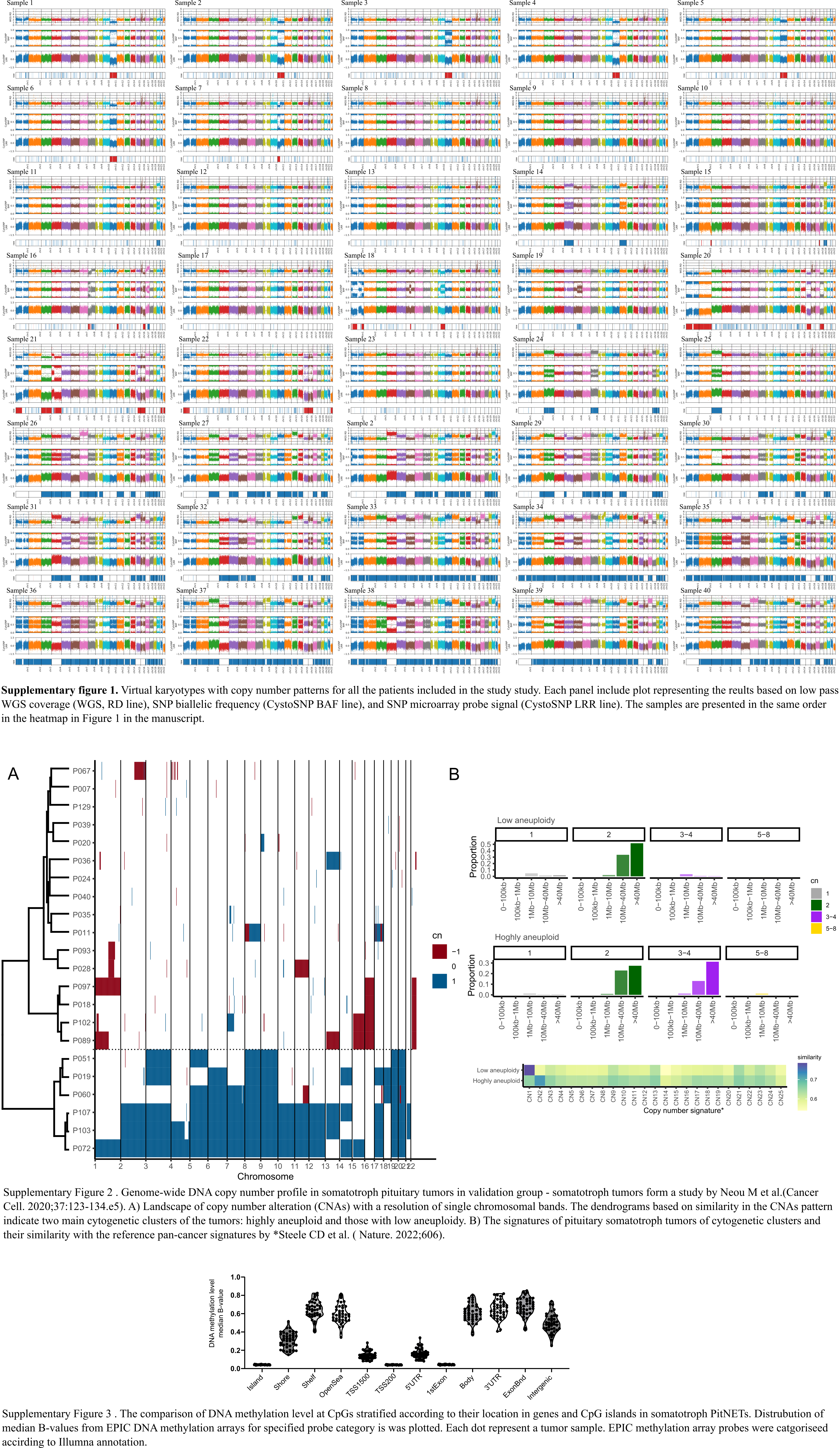

Supplement: Supplementary file 2 — Supplementary Material 2 [file 12967_2024_5736_MOESM2_ESM.png]
